# Women and ethnoracial minorities with poor cardiovascular health measures associated with a higher risk of developing mood disorder

**DOI:** 10.1186/s12911-021-01674-9

**Published:** 2021-12-24

**Authors:** Aixia Guo, Kari A. Stephens, Yosef M. Khan, James R. Langabeer, Randi E. Foraker

**Affiliations:** 1 Institute for Informatics (I2), Washington University School of Medicine, St. Louis, MO USA; 2 Family Medicine, University of Washington School of Medicine, Seattle, WA USA; 3 Health Informatics and Analytics, Centers for Health Metrics and Evaluation, American Heart Association, Dallas, TX USA; 4 School of Biomedical Informatics, Health Science Center at Houston, The University of Texas, Houston, TX USA; 5 Department of Internal Medicine, Washington University School of Medicine, St. Louis, MO USA

## Abstract

**Background:**

Mood disorders (MDS) are a type of mental health illness that effects millions of people in the United States. Early prediction of MDS can give providers greater opportunity to treat these disorders. We hypothesized that longitudinal cardiovascular health (CVH) measurements would be informative for MDS prediction.

**Methods:**

To test this hypothesis, the American Heart Association’s Guideline Advantage (TGA) dataset was used, which contained longitudinal EHR from 70 outpatient clinics. The statistical analysis and machine learning models were employed to identify the associations of the MDS and the longitudinal CVH metrics and other confounding factors.

**Results:**

Patients diagnosed with MDS consistently had a higher proportion of poor CVH compared to patients without MDS, with the largest difference between groups for Body mass index (BMI) and Smoking. Race and gender were associated with status of CVH metrics. Approximate 46% female patients with MDS had a poor hemoglobin A1C compared to 44% of those without MDS; 62% of those with MDS had poor BMI compared to 47% of those without MDS; 59% of those with MDS had poor blood pressure (BP) compared to 43% of those without MDS; and 43% of those with MDS were current smokers compared to 17% of those without MDS.

**Conclusions:**

Women and ethnoracial minorities with poor cardiovascular health measures were associated with a higher risk of development of MDS, which indicated the high utility for using routine medical records data collected in care to improve detection and treatment for MDS among patients with poor CVH.

**Supplementary Information:**

The online version contains supplementary material available at 10.1186/s12911-021-01674-9.

## Background

Mood disorders (MDS) are a type of mental health illness where the primary problem is a person’s abnormal changes in mood. MDS often present as chronic, waxing and waning conditions where mood issues, such as depression and anxiety, significantly cause distress or impairment in a person’s life [1]. MDS effects millions of people in the United States (U.S.) and MDS affects approximately 21.4% of U.S. adults across the lifespan [2]. In addition, approximately 20% of the U.S. population has suffered from depression in a given month and 12% of the population had two or more depressive episodes in a year [3].

Risk factors associated with MDS are varied. For example, some recent studies [4–6] reviewed and summarized risk factors for bipolar disorders, which included demographic (e.g., Black race, low education level) [7, 8], genetic (e.g., familial genetic risk, multiple SNPs) [9–13] and environmental risk factors (e.g., childhood trauma and brain injury, medical comorbidities, and obesity) [5, 12, 14–16]. Specific to depression, the following risk factors are commonly identified: demographics, cognitive processes, stressful life events and circumstances, and behavior patterns [17].

With respect to risk factors related to individual cardiovascular health (CVH) metrics [18], studies have failed to examine how each CVH metric contributes to MDS. Furthermore, many of these studies utilized single sites limiting generalizability and used traditional algorithms which were not optimized to study the specified associations.

In this study, The Guideline Advantage (TGA) clinical data registry established by the American Cancer Society, the American Diabetes Association, and the American Heart Association (AHA) was used to investigate the associations between longitudinal cardiovascular health measurements, other confounding factors, and the development of MDS. Also, machine learning methods were employed to predict the development of MDS using the time-series cardiovascular health CVH measures (i.e., smoking status (Smoking), body mass index (BMI), blood pressure (BP), glucose/hemoglobin A1c (A1C), and cholesterol [18]), age, gender, and race across a national sample of outpatient clinics. Using machine learning and deep learning methods across this national sample, several prediction models were explored to determine strength of CVH measures as predictors of MDS. Specifically, we compared random forest (RF) and logistic regression (LR) models with a deep learning algorithm called the long short-term memory (LSTM) model [19]. The LSTM can capture the informative and useful features and patterns in rich longitudinal EHR data [20, 21].

## Methods

### Data source

TGA is a clinical data registry established by the American Cancer Society, the American Diabetes Association, and the AHA, that was used to track and monitor disease management and outpatient preventative care [20, 22]. The TGA dataset was used in this study. A total of 37,667 unique patients were identified, diagnosed with MDS from the 362,533 patient cohort, of whom 8761 had more than 5 CVH measures in a 9-year period (2008–2016). A randomly selected cohort (n = 8869) of patients without MDS were also selected using the same criteria, for a total of 17,630 patients in the study population. Among these patients, 15,477 had at least one drug prescription (7996 patients with MDS and 7481 patients without MDS).

### CVH measurements

Five CVH measures were used: Smoking, BMI, BP, A1C, and cholesterol (low-density lipoprotein, LDL. Each of the five CVH measures were classified into one of three categories according (see Table 1): ideal, intermediate and poor. All drug names were converted to their corresponding drug class by using the Multum drug database [23] as a template. During the conversion process, the Levenshtein distance algorithm [24] was employed to compare drug names in the dataset with those in the Multum drug database. If the Levenshtein distance between compared strings was less than five, we considered the conversion valid and those medications were included in the analyses. All CVH measures and all medication orders prior to the date of MDS diagnosis were considered in the analysis for those who were diagnosed with MDS, and all CVH measures and all medications were considered in the analysis for those who were not diagnosed with MDS. All CVH measures and medication data were sorted in a time order.

**Table 1:** Measures of CVH which are available in the TGA (Adapted from: Lloyd-Jones, 2010) [25]

	Poor health	Intermediate health	Ideal health
Health behaviors			
Smoking status	Yes	Former ≤ 12 months	Never or quit > 12 months
Body mass index	≥ 30 kg/m^2^	25–29.9 kg/m^2^	< 25 kg/m^2^
Health Factors			
LDL	≥ 160 mg/dL	130–159 mg/dL or treated to goal	< 130 mg/dL
Blood pressure	Systolic ≥ 140 mm Hg or Diastolic ≥ 90 mm Hg	Systolic 120–139 mm Hg or Diastolic 80–89 mm Hg or treated to goal	Systolic < 120 mm Hg Diastolic < 80 mm Hg
Fasting plasma glucose	≥ 126 mg/dL	100–125 mg/dL or treated to goal	< 100 mg/dL

### Statistical analysis for association between gender, race, CVH, and MDS

The proportion of patients diagnosed with MDS among categories of gender and race was calculated. For each patient, if the patient had multiple diagnosis codes of MDS, the patient was counted once as having MDS. Otherwise, if a patient did not have any MDS diagnoses, then this patient was classified as not having MDS.

The proportion of patients who had poor CVH status on each metric between patients with and without MDS were compared. For each CVH metric, the patient was counted as having poor CVH if the patient ever had a measurement categorized as poor. For example, if a patient had two poor CVH measurements for A1C, then this patient was counted one time as having poor A1C. Otherwise, if a patient did not have any poor measurements for A1C, then this patient was not counted as having poor A1C. The denominator for each metric was the total unique number of patients with at least one measure of this targeted metric. The most prevalent medication classes for patients diagnosed with and without MDS was then investigated.

Moreover, the proportion of poor CVH status for each metric between patients with and without MDS based on different gender and race categories was also calculated. Specifically, when calculating the proportion of poor BMI among female patients with MDS, the number of female patients with MDS diagnosis and who had poor BMI was first calculated, then the denominator was the total number of female patients with an MDS diagnosis who also at least had one BMI measurement.

### Machine learning and deep learning for MDS prediction

To further investigate the possibility of MDS prediction using the longitudinal CVH metrics and other confounding factors. Three models, i.e., LSTM, RF, and LR, were employed to predict MDS from gender, race, CVH. To prepare CVH measures for the prediction in step 1, the metric name with its category was combined according to Table 1. For example, if a patient had a value for BP in the ideal category, then they were combined as bloodpressureideal. Then these measurements were mapped to a 32-dimensional vector space by word embedding [26]. For example, if a patient had a value for BP in the ideal category, then they were combined as bloodpressureideal. Then these measurements were mapped to a 32-dimensional vector space by word embedding technique Word2Vec. The Python Genism Word2Vec model was used with the following hyperparameters: size (embedding dimension) was 32, window (the maximum distance between a target word and all words around it) was 5, min_count (the minimum number of words counted when training the model) was 1, and sg (the training algorithm) was the continuous bag of words CBOW.

Each CVH measure was associated with a time point calculated by the difference in days between current date and the last visit date. Each CVH measure had its own time point. For example, one patient had two BP measures on 10/23/2015 and 11/23/2015, and one A1C measure on 09/23/2015, and the last or most recent date is 12/21/2015, then the time points for the two BP measures were 60 days and 30 days respectively, and the time point for A1C measure was 90 days. Moreover, each CVH measure was also associated with a measure outcome, i.e., ‘ideal’, ‘intermediate’ or ‘poor’. Thus, the longitudinal data of each patient was represented by the combination of the outcomes and time points of multiple CVH measures. For example, as shown in Fig. 5, one patient had multiple BMI measures in July 2011, March 2012, June 2012, October 2012, November 2012, and December 2012*.* Thus, each patient had its own vectors to represent their CVH measures, drug classes, or combined data in each step.

Next, the embedded patient vectors, plus gender, race, and age were fed to our LSTM models to investigate the associations between the outcome of MDS and longitudinal CVH measures, gender, and age. For the prediction, the dataset was randomly split into a training dataset (80%), validation dataset (10%), and testing dataset (10%). The models were trained in a training data set, validated in the validation dataset, and then applied to a test data set. The area under the receiver operator characteristic (ROC) curve (AUC) and other metrics, including: accuracy, precision, recall, specificity, and F1-score, were used to evaluate the performance of the models.

### Case study for model interpretation

For the LSTM model, a case study analysis was conducted to better understand the mechanism of model prediction. Specifically, for a randomly selected a patient with MDS, one CVH metric feature were dropped at each time to determine the influence of that CVH metric, and the resulting new probability was compared to the original prediction probability (belongting to the MDS). For gender and race importance analysis, we imputed the value of this factor to a non-meaningful value to compare the resulting probability with the original. Analyses were conducted using the libraries of Scikit- learn, Keras, Scipy, and Matplotlib with Python, version 3.6.5, in 2020.

## Results

### Characteristics of the overall study population

Our study population varied across demographics for patients with and without MDS (see Table 2). Most patients were (61%) female, 50% white, with an average age of 40 years. The average value of A1C was 7.2%, while that of LDL was 108 mg/dL, BMI was 29.4 kg/m^2^, systolic BP (BPS) was 123 mmHg and diastolic BP (BPD) was 74 mmHg. Approximately 25% of patients were current smokers. Among patients with MDS, the average value of A1C was 7.3%, while that of LDL was 112 mg/dL, BMI was 31.6 kg/m^2^, BPS was 124 mmHg, and diastolic BP (BPD) was 76 mmHg. Approximately 37% of patients were current smokers.

**Table 2:** Characteristics [mean, (SD) or n (%)] of the overall study population

Patients demographics and CVH measures	Total patients	Patients with MDS	Patients with no MDS	P-value
Number of patients	17,630	8761	8869	
*Demographics*				
Age (years), Mean (SD)	40 (23)	42 (19)	38 (26)	< 0.001
Race n (%)				
White	8822 (50.0)	4458 (50.9)	4364 (49.2)	0.03
Black	1049 (6.0)	357 (4.1)	692 (7.8)	< 0.001
Other	1543 (8.8)	621 (7.1)	922 (10.4)	< 0.001
Unknown	6264 (35.5)	3355 (38.3)	2909 (32.8)	< 0.001
Gender n (%)				
Female	10,737 (60.9)	5971 (68.2)	4766 (53.8)	< 0.001
Male	6888 (39.1)	2789 (31.8)	4099 (46.2)	< 0.001
Other	5 (0)	1 (0.0)	4 (0.0)	
*CVH measures Mean (SD)*				
A1C (%)	7.2 (1.9)	7.3 (1.9)	7.1 (1.8)	0.12
LDL (mg/dL)	107.5 (36.3)	111.5 (36.7)	104.2 (35.6)	0.033
BMI (kg/m^2^)	29.4 (9.3)	31.6 (9.3)	27.6 (8.9)	< 0.001
BPS (mmHg)	123 (19)	124 (18)	122 (20)	< 0.001
BPD (mmHg)	74 (15)	76 (12)	72 (17)	< 0.001
Smoking *n (%)*	4390 (24.9)	2906 (36.9)	1484 (16.7)	< 0.001
Overweight and obese	7451 (42.3)	3467 (81.3)	3984 (68.5)	< 0.001
Hypertension	7985 (45.3)	4222 (48.2)	3763 (42.4)	< 0.001

### Association between gender, race and MDS

**Fig. 1:**
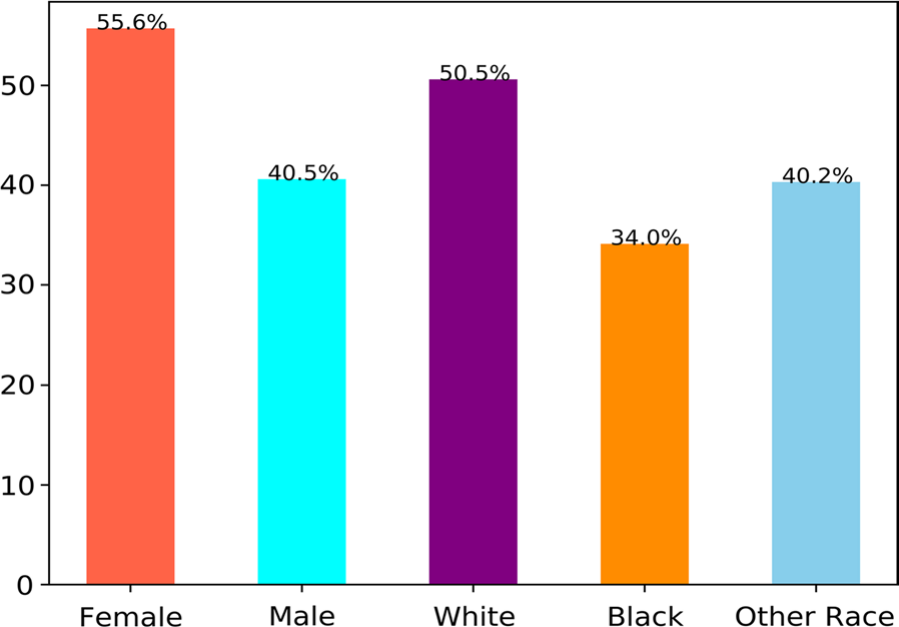
Proportion of patients with MDS based on gender and race

Among 10,737 female patients, 5971 (55.6%) were diagnosed with MDS. There were 4458 (50.5%) white patients diagnosed with MDS among all 8822 white patients. Among 1049 of black race, 357 (34%) patients had a diagnosis of MDS. The results indicated that the proportion of patients with MDS differed according to gender and race (see Fig. 1). The Fisher’s exact test results indicated Female group and Male group was significantly different with MDS (P < 0.001). Patients with white race and patients with black race were statistically significantly different with P < 0.001.

### Association between CVH health metrics and MDS

**Fig. 2:**
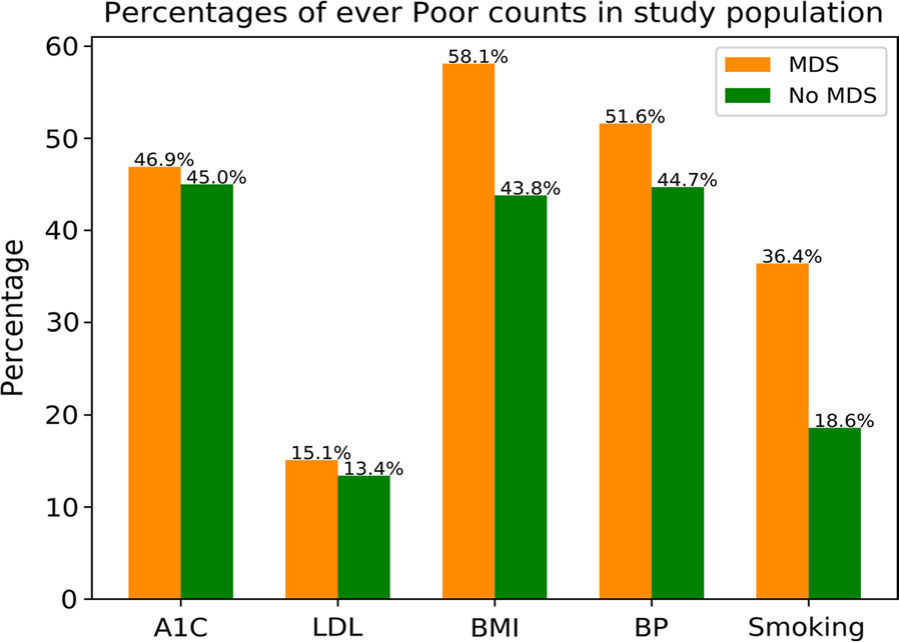
Poor status (n, %) of each CVH metric among patients diagnosed with MDS and among those without MDS

Patients diagnosed with MDS consistently had a higher proportion of poor CVH compared to patients without MDS, with the largest difference between groups for BMI and Smoking (see Fig. 2). Among patients with at least one A1C measure, 47% patients with MDS had poor A1C compared to 45% of patients without MDS. Similar trends were observed among the other four metrics: approximately 15% of patients with MDS had poor LDL (58% for BMI, 52% for BP, and 36% for Smoking), while 13% patients without MDS had a poor LDL (44% for BMI, 45% for BP, and 19% for Smoking). Results of Fisher’s exact test for each CVH metric showed that LDL (P = 0.033), BMI (P < 0.001), BP (P < 0.001), and Smoking (P < 0.001) were significantly associated with outcome of MDS, while A1C (P = 0.11) was not significantly different.

### Association between gender, race, CVH, and MDS

**Fig. 3:**
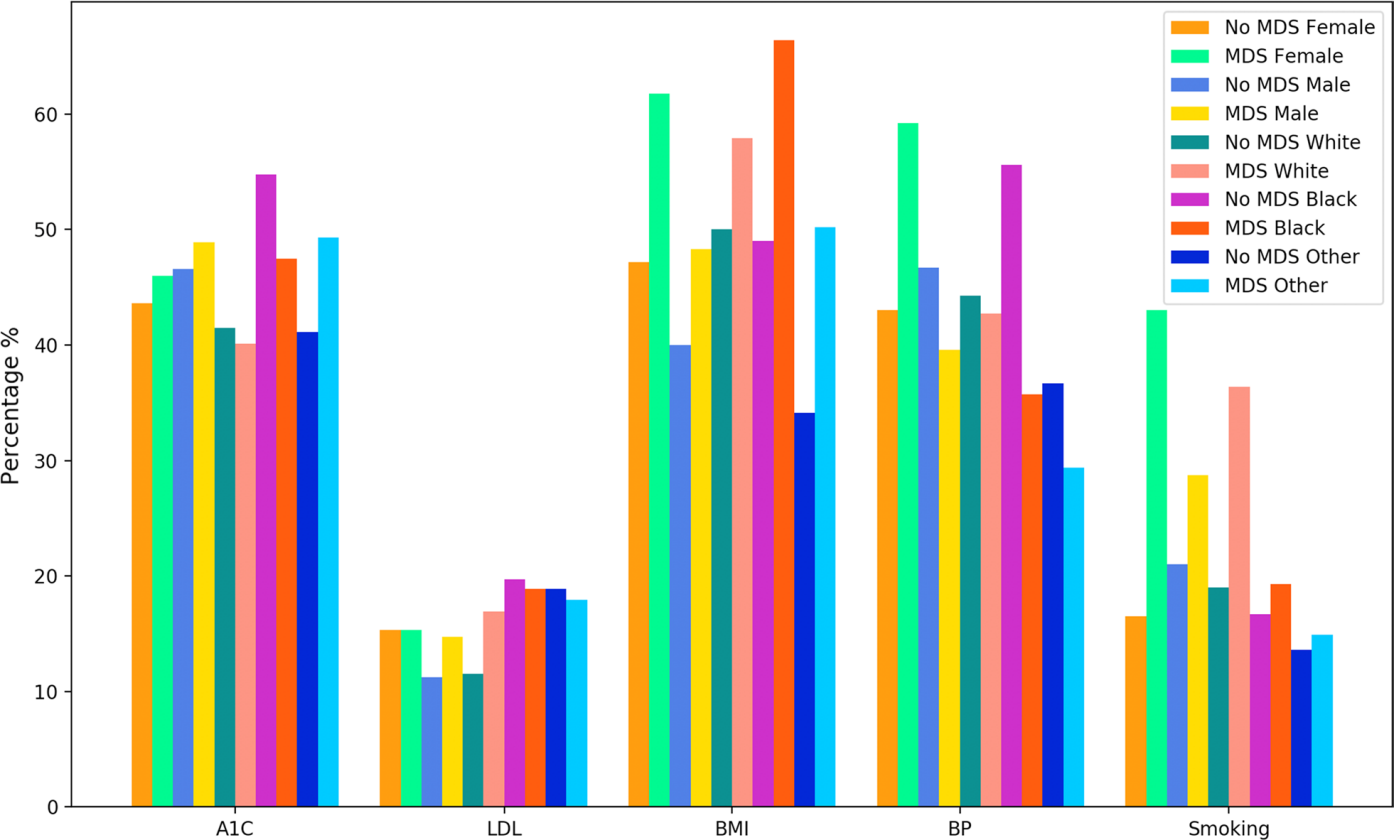
Poor status (%) of each CVH metric among those diagnosed with MDS and those without MDS according to gender and race strata.

Race and gender were associated with varying rates of poor status of CVH metrics (see Fig. 3). Among female patients, 46% of those with MDS had a poor.

A1C compared to 44% of those without MDS; 62% of those with MDS had poor BMI compared to 47% of those without MDS; 59% of those with MDS had poor BP compared to 43% of those without MDS; and 43% of those with MDS were current smokers compared to 17% of those without MDS. In addition, black patients with MDS had worse BMI (66%), followed by female patients (62%), and white patients with MDS (58%) as compared to those without MDS. We summarized the P-values to the Table S1 in the Additional file 1.

### Prediction of MDS using CVH metrics, gender, race and age via machine learning and deep learning models

**Fig. 4:**
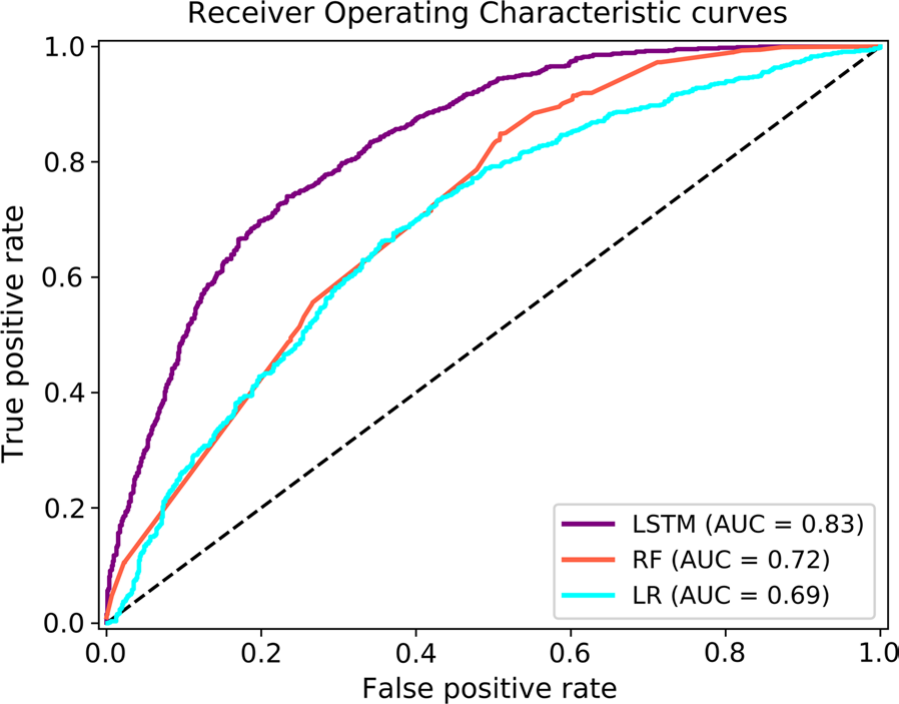
Area under the curve (AUC) evaluation of model performance by LSTM, RF, and LR models by using predictors of CVH, age, race, and gender

Prediction by LSTM was superior to the other models with an AUC of 0.83. Meanwhile, the AUC for prediction by RF was 0.72, and the AUC for prediction by LR was 0.69 (see Fig. 4). The accuracy was 0.75 for LSTM, 0.67 for RF, and 0.65 for LR. The F1-score was 0.77 for LSTM, 0.73 for RF, and 0.66 for LR. The LSTM achieved the best performance based on the F1-score (see Table 3). The cut-off of risk prediction of development of MDS was 0.5 for calculating these metrics.

**Table 3:** Other more metrics to evaluate the model performance

Models	Accuracy	Precision	Recall	Specificity	F1-score
LSTM	0.75	0.79	0.82	0.68	0.77
RF	0.67	0.62	0.88	0.45	0.73
LR	0.65	0.64	0.68	0.63	0.66

### Case study for model interpretation of LSTM

**Fig. 5:**
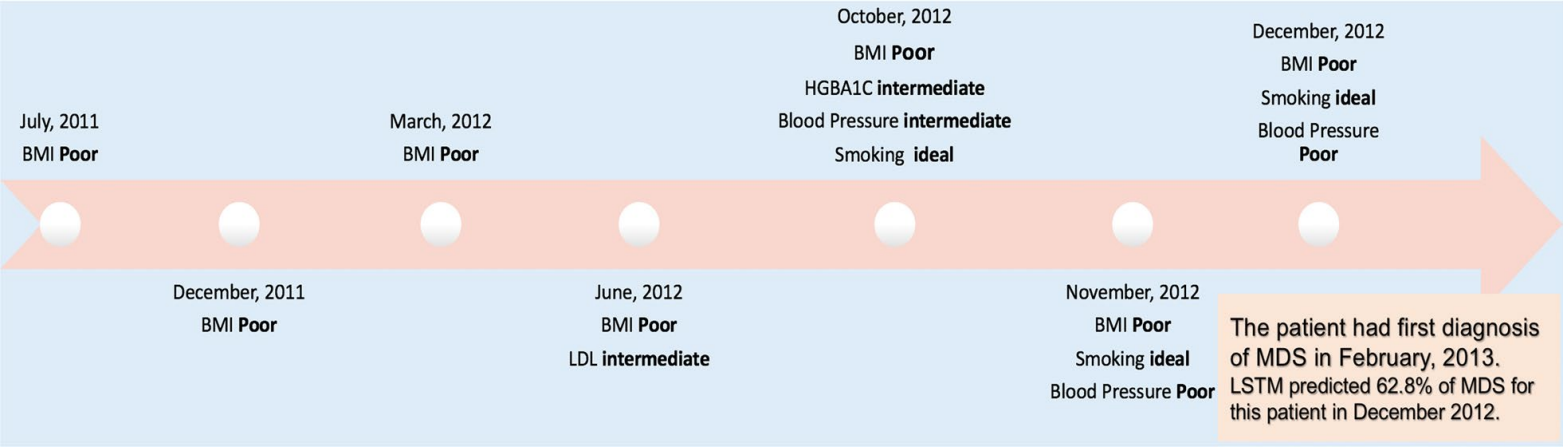
Illustration of a random patient example of prediction by LSTM model. The record of a 47-year old female patient with white race showed poor longitudinal BMI and BP. The timeline of measures is shown in ascending order

We demonstrated the feature importance for a given patient's prediction. The randomly selected patient was white female and was diagnosis with MDS. The deep learning model predicted the true positive of risk probability of developing MDS for this patient was 62.8%. For the demonstration, we show the top features/measures in a time ascending order, as determined by the deep learning model (see Fig. 5). The patient had 16 longitudinal CVH metric measures before the time diagnosis of MDS. For the CVH measures, the model identified poor BMI as the most important feature, followed by poor BP, intermediate BP, LDL, and A1C. We found that the LSTM model also considered gender and race as important features. These discriminatory factors were identified by the deep learning model, not by manual selection.

## Discussion

In this study, the association analyses between CVH metrics and other confounding factors were conducted by using 9-year longitudinal EHR data from 70 different outpatient clinics in the US. The analysis results indicated that patients diagnosed with MDS had a higher prevalence of poor CVH compared to those who were not diagnosed with MDS. The female and black patients with poor CVH health metrics are at a higher risk associated with MDS development. Patients with MDS diagnosis and with a black race had the worst BMI measurements. In another word, women and ethnoracial minorities with poor cardiovascular health measures were associated with a higher risk of developing mood disorder.

Clinical diagnosis and characterization of MDS are nontrivial, which require labor-intensive and longitudinal diagnostic interviews and evaluation of the medical history of individual patients. The availability of EHR data, analyzed by machine learning models, can facilitate the diagnosis and phenotype characterization of MDS [27]. However, the predictive and causal phenotype and biomarkers of MDS remain unclear. Recent studies indicated some common genetic biomarkers, e.g., inflammation and immune related genetic biomarkers, between MDS and CVH [28–30]. In addition to the genetic associations, in this study, we conducted association analysis between MDS and CVH using a unique and large TGA data set which contained longitudinal EHR data of CVH measures and patient demographics from 70 different outpatient clinics across the U.S.

Moreover, the MDS prediction using machine learning and deep learning models showed that the longitudinal CVH measures and other confounding factors are informative to predict the development of MDS. The LSTM model outperformed other machine learning models. In addition, techniques for sensitivity analysis by directly perturbing features representing records of CVH measurements could aid in the discovery of important features in the general population of patients [31]. To determine which features are important to intervene upon in advance, the feature importance can be conducted for individual patients (see Fig. 5).

Our study used cut-offs in Table 1 for BP category, which were slightly different from the most recent BP guidelines [32]. The ideal category would not affect as most recent cut-off for ideal is the same as we used, while intermediate and poor cut-offs lowered compared to the ones in Table 1. It may result in more poor cases from intermediate cases, which may slightly affect results presented in the manuscript as there were relatively very few cases of intermediate cases in our manuscript [33].

Our study data source did not collect and include diet and physical activity (PA) data for the patients that are included in the Life’s Simple 7. Incorporating diet and PA data will make our study more complete and further improve machine learning prediction accuracy.

In the future work, we aim to investigate the associated or causal and CVH related diagnostic phenotypes, using the longitudinal medical records, that occurred before the diagnosis of MDS, and are informative for the MDS prediction. These diagnostic phenotypes can be important for treatment decision-making to prevent MDS. Moreover, it is interesting to investigate the associations between genetic biomarkers and phenotypes that are related to MDS and CVH.

One advantage of our analysis was the unique TGA data set which contained longitudinal EHR data of CVH measures and patient demographics from 70 different outpatient clinics across the US. To our knowledge, this was the first systematical analysis to investigate the association between the development of MDS and the combined data of all five longitudinal CVH risk factors with gender and race. If a patient was predicted to have a high probability of developing MDS in a year, then the patient and providers could better maintain or control the CVH measures to prevent the patient from developing a MDS. During the process of maintaining and controlling better CVH levels, patients might also have better outcomes with respect to with lower incidence of cardiovascular disease and cancers [34–38]. Thus, predicting future development of MDS can indirectly decrease the cost and burden on the health system caused by major chronic diseases.

## Limitations

A limitation to our study was that patients in our data set had different numbers of CVH measurements. We excluded patients who had too few CVH measurements (four or fewer), which may impact the generalizability of our results.

## Conclusions

In this study, our analysis results indicated that women and ethnoracial minorities with poor cardiovascular health measures were associated with a higher risk of developing mood disorder, which indicated the high utility for using routine medical records data collected in care to improve detection and treatment for MDS among patients with poor CVH.

## Supplementary Information

**Additional file 1.** Table S1. P-values of gender and race groups by Fisher’s exact test.

## Data Availability

The datasets belong to a third-party and the authors do not have permission to share the data. Researchers need to apply from American heart association for the access to the datasets.
