# Supplementary material for: Women and ethnoracial minorities with poor cardiovascular health measures associated with a higher risk of developing mood disorder
Source: BMC Med Inform Decis Mak. 2021 Dec 24;21:361. doi: 10.1186/s12911-021-01674-9 (PMC8709948; doi:10.1186/s12911-021-01674-9)
Supplement: Supplementary file 1 — Additional file 1. Table S1. P-values of gender and race groups by Fisher’s exact test. [file 12911_2021_1674_MOESM1_ESM.docx]

**Supplementary Material**

**Table S1. P-values of gender and race groups by Fisher’s exact test.**

|  | A1C | LDL | BMI | BP | Smoking |
| --- | --- | --- | --- | --- | --- |
| Female vs Male | 7.95*10^-31^ | 0.10 | 2.33*10^-215^ | 1.29*10^-47^ | 1.21*10^-66^ |
| White vs Black | 4.92*10^-33^ | 7.58*10^-12^ | 1.64*10^-41^ | 1.72*10^-52^ | 2.25*10^-5^ |
| White vs Other or Unknown | 0.0002 | 0.002 | 0.0 | 3.01*10^-22^ | 0.32 |
| Black vs Other or Unknown | 3.81*10^-24^ | 8.02*10^-7^ | 1.72*10^-20^ | 1.41*10^-95^ | 9.06*10^-5^ |
